# Psychological Distress Trajectories in Residential Alcohol and Other Drug Treatment

**DOI:** 10.1111/dar.14099

**Published:** 2025-06-17

**Authors:** Emma L. Hatton, Peter J. Kelly, Laura Robinson, Alison Beck, Mei L. Lee, Robert Stirling, Lauren Mullaney, Michele Campbell, Briony Larance

**Affiliations:** ^1^ School of Psychology University of Wollongong Wollongong Australia; ^2^ Network of Alcohol and Other Drug Agencies Sydney Australia; ^3^ Drug Policy Modelling Program Social Policy Research Centre, UNSW Sydney Sydney Australia; ^4^ Triple Care Farm, Mission Australia Knights Hill Australia

## Abstract

**Introduction:**

Residential clients frequently report high psychological distress at intake, but little is known about changes in distress throughout treatment. This study aimed to identify in‐treatment trajectories for psychological distress and factors associated with trajectory classes.

**Methods:**

A retrospective cohort of adults attending Australian non‐government residential substance use treatment between 2012 and 2023 was identified from routinely collected data, NADAbase. Participants (*N* = 1492) completed ≥ 3 Kessler‐10 Psychological Distress Scale (K10) assessments within 90 days of intake. Latent growth curve analyses identified classes of K10 trajectories. Multinomial regression identified demographic and clinical correlates (Severity of Dependence Scale [SDS] and EUROHIS Quality of Life scale [EQoL‐8]) of class membership.

**Results:**

A five‐class model describing K10 trajectories (1: moderate–low improved; 2: high–low improved; 3: very high–moderate improved; 4: very high–high improved; 5: very high unchanged) had the best model fit. Compared to high–low improved (34.5%; referent), moderate–low improved (45.4%) were less likely to identify as female, have higher SDS and lower EQoL‐8 scores, or use cannabis; very high–moderate improved (13.1%) were more likely to have lower SDS scores, be aged under 25 and use opioids; very high–high improved (5.6%) were less likely to identify as male, be aged over 25, have higher EQoL and SDS scores; and very high unchanged (1.3%) were more likely to have lower EQoL scores and have left without completing treatment.

**Discussion and Conclusions:**

Four K10 trajectory classes showed improvement after 90 days. Around 7% reported sustained high to very high psychological distress. Routine monitoring of psychological distress provides opportunities to identify non‐improving clients and review treatment plans to improve outcomes.

**Summary**

- Five psychological distress trajectories were found during residential treatment.
- Four classes showed improvement in psychological distress within the first 90 days.
- Seven percent of residential clients had persistent high to very high psychological distress.

## 1 Introduction

Residential alcohol and other drug (AOD) treatment combines intensive care with evidence‐based interventions for individuals with moderate to severe substance use disorders and associated issues [1, 2, 3]. These interventions include cognitive behavioural therapy (CBT), individual and group therapy and therapeutic community approaches where peers act as change agents [2]. Psychological distress is predominantly high for all residential treatment attendees at intake [4, 5, 6] and is higher for treatment non‐completers [7, 8].

Psychological distress refers to an emotional state involving discomfort associated with stressors, that result in fluctuations of mood and symptoms of emotion or physical harm [9, 10]. This construct has five key attributes: “(1) perceived inability to cope effectively, (2) change in emotional status, (3) discomfort, (4) communication of discomfort and (5) harm” [10]. Higher psychological distress is associated with loss of interest, low mood, hopelessness and restlessness [11] and can indicate a psychological disorder, such as depression and anxiety [10], which commonly co‐occur with substance use disorders [12, 13]. Systematic reviews suggest integrated treatment combining substance use and mental health interventions in residential services are best practice [1, 2, 14, 15]. It is essential that people attending residential AOD treatment are adequately supported to manage their levels of psychological distress, as this can be a contributing factor for ongoing substance use or relapse [16, 17].

Most studies assessing mental health outcomes, like psychological distress, in residential AOD treatment predominantly focus on pre–post or after discharge treatment outcomes and report significant improvements [2]. Similar improvements have been demonstrated in change scores for substance use and social outcomes like dependence severity and quality of life [5, 6, 18]. However, change scores only show variation between two time points or for pre‐determined groups. More sophisticated analytical models are needed to explore additional time points and identify data‐driven groups.

Previous studies have explored group trajectories in psychological distress during and after various AOD treatment using Generalised Estimating Equations [19], Linear Mixed Models [17] and latent class growth analysis [4]. Differences in trajectories have been identified for drug types [19] and abstinence stability [17]. In a sample of Australian men (*N* = 624) attending New South Wales (NSW) Aboriginal residential AOD services, Chambers et al. [4] identified four classes of psychological distress. Two classes started with higher psychological distress and decreased over time (37% and 34%), one class had mostly high psychological distress but variable trajectories (16%), and the final class had stable low psychological distress scores (13%). Unstable housing and precarious income were associated with initial and persisting higher psychological distress trajectories, and being aged under 35 years was associated with a lower likelihood of low psychological distress trajectories.

The use of latent class approaches, like in Chambers et al. [4], is beneficial to finding these underlying patterns of psychological distress among residential treatment clients. These modelling approaches cluster individuals based on data‐driven similarities with rigorous criteria, allowing comparisons for model fit and number of classes [20]. When assessing trajectories or change over time, latent class growth analyses are a recommended method for identifying subgroup classifications in longitudinal data [21]. More research is needed to establish group differences in psychological distress within residential treatment for people attending non‐Indigenous services, including women. Evaluating these relationships in treatment enables further discussion with clients around treatment progress, goals and additional support as part of their care where needed. This study used latent curve growth analysis to examine routinely collected data from residential AOD treatment services to:

— Explore whether groups of clients can be identified according to their specific psychological distress trajectories over time.
— Examine the associations between group membership (based on psychological distress trajectories) and key demographic and clinical characteristics.

## 2 Methods

### 2.1 Study Design

A retrospective cohort design was used. The current study received approval from the University of Wollongong Human Research Ethics Committee (HE12/207) and was endorsed by the Community Mental Health, Drug & Alcohol Research Network Research Ethics Consultation Committee to conduct analyses and reporting of non‐identifiable data.

### 2.2 NADAbase

The Network of Alcohol and other Drug Agencies (NADA) is the peak body representing non‐government organisations within the AOD sector in NSW. Since 2010, over 100 NADA member services routinely collect mandatory treatment data [22, 23] and some additionally collect a standard set of routine outcome measures (Client Outcomes Management System, COMS; [20, 24]) within the NADA electronic database (NADAbase). This data is de‐identified upon extraction for use in research.

NADAbase is designed to capture outcome data across treatment. Common timepoints include intake, 30 or 60 days; however, the actual timing of administration is determined by individual services. Therefore, between services, there is variability in timing and frequency of outcome data. Outcome data for psychological distress, quality of life and Severity of Dependence from the COMS were grouped into time‐period categories arranged to describe discrete one‐month periods: (i) intake (1–14 days); (ii) 30 days (15–44 days); (iii) 60 days (45–74 days); and (iv) 90 days (75–104 days), following the guidelines suggested in previous NADAbase research [18]. Clients who completed three assessments would need to have attended treatment for a minimum of 45 days to complete intake, 30 and 60 days assessments.

### 2.3 Participants

**FIGURE 1:**
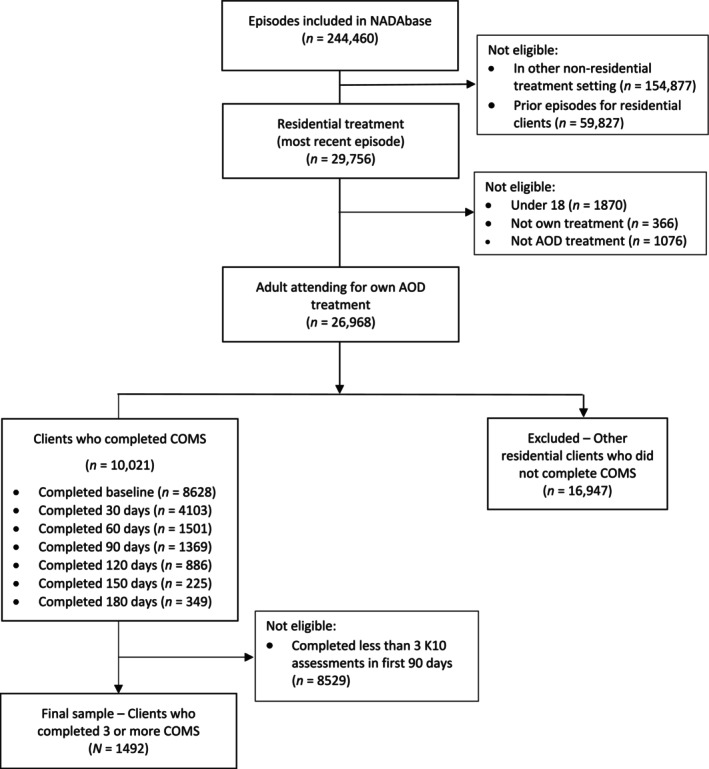
CONSORT diagram. AOD, alcohol and other drugs; COMS, Client Outcomes Management System; K10, Kessler‐10 Psychological Distress Scale.

Eligibility for inclusion in the study was based on demographic and treatment characteristics from the NADAbase. Participants needed to have: (i) attended residential treatment for their own AOD use between February 2012 and May 2023; (ii) been aged over 18 years at treatment entry; and (iii) completed at least three COMS assessments between intake and the 90 days assessment [25]. Data was analysed for the most recent treatment episode, as some clients had multiple residential treatment episodes in NADAbase. Of 5011 clients who attended treatment for a sufficient duration to complete three COMS assessments, the final sample for the trajectory analyses consisted of 1492 (29.8%) participants. Participants were attendees from 37 different residential rehabilitation services with a variety of treatment models and service‐level characteristics. For comparison of the final sample to other residential clients in NADAbase, see Supporting Information: Table A1. See Figure 1 for CONSORT diagram.

### 2.4 Measures

#### 2.4.1 Demographics

Demographics included: age (under 25 years/25 years and over), described gender (male/female/other), principal source of income, living arrangement, usual accommodation and principal drug of concern. Principal drug of concern was coded into stimulants, alcohol, cannabis, heroin, other opioids and other drugs based on the Australian Standard Classification of Drugs of Concern [26].

#### 2.4.2 Psychological Distress

The Kessler‐10 Psychological Distress Scale (K10; [27, 28, 29]) consists of 10 items assessing psychological distress in the prior 4 weeks. Items (e.g., ‘About how often did you feel hopeless?’) were scored from (1) ‘*none of the time*’ to (5) ‘*all of the time*’ on a Likert scale. Totals scores range from 10 to 50 and can be grouped into clinical categories with ranges of 10–19 indicating low, 20–24 indicating moderate, 25–29 indicating high and 30–50 indicating very high, psychological distress [29, 30, 31].

#### 2.4.3 Dependence Severity

The Severity of Dependence Scale (SDS; [32]) is five‐item Likert scale measuring psychological components of dependence, including impaired control, preoccupation or anxiety around substance use. This scale records substance of ‘greatest concern’ and levels of dependence on this substance. Items (e.g., ‘Did you worry about your use of (drug)’) were scored from (0) ‘*never or almost never*’ to (3) ‘*always or nearly always*’. Scores range from 0 to 15, with higher scores indicating greater dependence severity.

#### 2.4.4 Quality of Life

The EUROHIS‐Quality of Life (EQoL‐8; Schmidt et al. [33]) index is an eight‐item measure, assessing quality of life within the previous 2 weeks, was developed as a short‐version of the WHOQOL‐BREF and WHOQOL‐100 [34, 35, 36]. Participants rated items (e.g., ‘How would you rate your quality of life?’) on a five‐point Likert Scale from (1) ‘*not at all*’ or ‘*very dissatisfied*’ to (5) ‘*completely*’ or ‘*very satisfied*’. Scores range from 8 to 40 with higher scores indicating higher quality of life.

### 2.5 Statistical Analyses

Baseline demographic characteristics were explored in SPSS Version 28 (32). Latent class growth analyses were used in Mplus (Version 8.5) [37] to identify the number of latent trajectories for psychological distress across 3–4 timepoints. Missingness was addressed in Mplus using a full‐information maximum likelihood estimation under the missing at random assumption [38]. Mplus provides a matrix of covariance coverage which demonstrates the extent of missingness in the available observations for individual and pairs of variables. Values greater than 0.10 are necessary for model convergence [39]. For the current study, coverage ranged from 0.26 to 0.98.

Beginning with a single class model, the number of classes was increased by one for each model until optimum model fit was found. Model fit was assessed using multiple fit indices including: the Bootstrap Likelihood Ratio Test, Bayesian Information Criterion, Lo–Mendell–Rubin Likelihood Ratio Test, trajectory class size including more than 1% of the total sample [38], high entropy and high posterior probabilities. For the Bootstrap Likelihood Ratio Test, a significant *p* value indicates better fit for the *k* class model than the model with one fewer class (*k*−1 class model) [38]. For entropy and posterior probabilities, values approaching 1.0 indicate appropriate model fit [38]. Classes were labelled according to K10 clinical categories at intake and 90 days assessment [30].

Multinomial logistic regression using forward stepwise selection in SPSS was used to examine the intake demographic and clinical correlates of class membership. Categories for demographics were collapsed or removed where less than 5% of the total sample were included in subgroups. Class 2 (high–low improved) was treated as the referent category. Relative risk ratios and 95% confidence intervals for each trajectory class comparison to the referent category were reported. Sensitivity analyses using chi‐square tests, Kruskal–Wallis tests and post hoc tests were conducted to compare the final sample to other NADAbase clients who completed less than three COMS assessments or were not a COMS client (see Supporting Information: Appendix A). Descriptive statistics and independent *t*‐tests of the COMS sample were conducted to compare treatment duration for clients who had left treatment early or completed their planned treatment episode to identify risk points for early/unplanned treatment cessation (see Supporting Information: Appendix B). The reasons for treatment cessation and psychological distress for participants not included in the final sample were also compared in cross‐tabulation (see Supporting Information: Appendix B).

## 3 Results

### 3.1 Sample Demographics

The total sample was majority male‐identifying (71.6%), Australian‐born (90.2%) and had stable usual accommodation (77.9%). Participant ages ranged from 18 to 72 years at intake (*M* = 36.72, SD = 9.60) years. Length of stay for the residential rehabilitation episode averaged 174.92 days (SD = 116.73). Participants most reported stimulants (46.1%) as the primary drug of concern, followed by alcohol (34.6%), heroin (11.5%), cannabis (7.9%), other opioids (2.2%) and other drugs (2.2%). See Table 1 for all baseline demographic characteristics and treatment outcomes. Comparisons between the current sample and excluded residential clients in the NADAbase (Supporting Information: Appendix A) found the samples had some differences but were mostly comparable and representative of NSW residential AOD clients.

**TABLE 1:** Baseline demographics and psychological distress, Severity of Dependence and quality of life at baseline, 30, 60 and 90 days by psychological distress trajectory class membership (*N* = 1492).

	Total sample (*N* = 1492)	Class 1: Moderate–low improved (*n* = 667; 45.4%)	Class 2: High–low improved (*n* = 515; 34.4%)	Class 3: Very high–moderate improved (*n* = 196; 13.1%)	Class 4: Very high–high improved (*n* = 84; 5.6%)	Class 5: Very high unchanged (*n* = 20; 1.3%)
Baseline demographics						
Age, years^a^	36.72 (9.60)	37.50 (9.41)	36.72 (9.63)	32.24 (9.50)	34.88 (10.84)	32.79 (7.69)
Described gender						
Men	1068 (71.6)	530 (78.3)	351 (68.2)	130 (66.3)	45 (53.6)	12 (60.0)
Women	409 (27.4)	144 (21.3)	157 (30.5)	64 (32.7)	37 (44.0)	7 (35.0)
Other	15 (1.0)	≤ 5 (0.4)	7 (1.4)	≤ 5 (1.0)	≤ 5 (2.4)	≤ 5 (5.0)
Principal source of income						
Benefits/pension/retirement fund	1297 (86.9)	588 (86.9)	440 (85.4)	177 (90.3)	75 (89.3)	17 (85.0)
No income/dependent on others	57 (3.8)	26 (3.8)	19 (3.7)	≤ 5 (2.6)	6 (7.1)	≤ 5 (5.0)
Employment	101 (6.8)	47 (6.9)	41 (8.0)	9 (4.6)	≤ 5 (2.4)	≤ 5 (10.0)
Other/not known	37 (2.5)	16 (2.4)	15 (2.9)	≤ 5 (2.6)	≤ 5 (1.2)	—
Living arrangement						
Friend(s)/other relative	717 (48.1)	323 (47.7)	258 (50.1)	94 (48.0)	31 (36.9)	11 (55.0)
Alone	383 (25.7)	157 (23.2)	138 (26.8)	53 (27.0)	30 (35.7)	≤ 5 (25.0)
Spouse/partner +/− child(ren)	203 (13.6)	113 (16.7)	56 (10.9)	23 (11.7)	8 (9.5)	≤ 5 (15.0)
Other/not stated	130 (8.7)	58 (8.6)	41 (8.0)	19 (9.7)	11 (13.1)	≤ 5 (5.0)
Alone with child(ren)	59 (4.0)	26 (3.8)	22 (4.3)	7 (3.6)	≤ 5 (4.8)	≤ 5 (10.0)
Usual accommodation						
Stable housing	1163 (77.9)	544 (80.4)	395 (76.7)	147 (75.0)	62 (73.8)	15 (75.0)
Unstable housing	217 (14.5)	82 (12.1)	84 (16.3)	33 (16.8)	14 (16.7)	≤ 5 (20.0)
Treatment facility/hospital	44 (2.9)	24 (3.5)	10 (1.9)	9 (4.6)	≤ 5 (1.2)	—
Other/not stated	40 (2.7)	12 (1.8)	18 (3.5)	6 (3.1)	≤ 5 (4.8)	—
Prison or detention centre	28 (1.9)	15 (2.2)	8 (1.6)	≤ 5 (0.5)	≤ 5 (3.6)	≤ 5 (5.0)
Country of birth						
Australia	1346 (90.2)	604 (89.2)	468 (90.9)	178 (90.8)	77 (91.7)	19 (95.0)
Other	146 (9.8)	73 (10.8)	47 (9.1)	18 (9.2)	7 (8.3)	≤ 5 (5.0)
Substance use						
Principal drug of concern						
Stimulants	621 (41.6)	317 (46.8)	193 (37.5)	77 (39.3)	29 (34.5)	≤ 5 (25.0)
Alcohol	516 (34.6)	219 (32.3)	198 (38.4)	61 (31.1)	31 (36.9)	7 (35.0)
Heroin	171 (11.5)	79 (11.7)	49 (9.5)	33 (16.8)	7 (8.3)	≤ 5 (15.0)
Cannabis	118 (7.9)	38 (5.6)	49 (9.5)	16 (8.2)	11 (13.1)	≤ 5 (20.0)
Other opioids	33 (2.2)	12 (1.8)	11 (2.1)	6 (3.1)	≤ 5 (4.8)	—
Other	33 (2.2)	12 (1.8)	15 (2.9)	≤ 5 (1.5)	≤ 5 (2.4)	≤ 5 (5.0)
Treatment characteristics						
Treatment episode duration^a^	174.92 (116.73)	169.92 (114.22)	183.76 (112.40)	174.33 (114.96)	170.42 (163.52)	141.15 (81.18)
Reason for cessation						
Completed treatment	1266 (84.9)	594 (87.7)	443 (86.0)	159 (81.1)	62 (73.8)	8 (40.0)
Left treatment early	194 (13.0)	75 (11.1)	63 (12.2)	31 (15.8)	16 (19.0)	9 (45.0)
Other	32 (2.1)	8 (1.2)	9 (1.7)	6 (3.1)	6 (7.1)	< 5 (15.0)
Treatment outcomes						
Quality of Life (EQoL‐8)^a^						
Baseline	25.17 (6.72)	27.47 (6.43)	23.72 (6.23)	23.26 (6.71)	21.53 (5.58)	18.30 (5.62)
30 days	31.41 (5.09)	33.65 (4.07)	30.63 (4.61)	28.69 (4.61)	26.16 (5.30)	21.22 (6.54)
60 days	32.31 (5.19)	34.63 (4.08)	31.52 (4.44)	29.38 (4.47)	26.75 (5.53)	21.75 (7.75)
90 days	33.73 (4.72)	36.29 (3.10)	33.55 (3.79)	30.95 (3.58)	26.92 (5.75)	21.21 (5.25)
Severity of Dependence (SDS)^a^						
Baseline	9.49 (3.68)	8.85 (3.77)	10.36 (3.29)	9.54 (3.83)	9.18 (3.56)	10.05 (4.39)
30 days	8.57 (3.92)	7.94 (4.05)	9.36 (3.68)	8.97 (3.73)	9.32 (3.62)	10.17 (4.30)
60 days	7.05 (4.27)	6.25 (4.25)	7.57 (4.28)	8.42 (3.96)	7.58 (3.65)	9.50 (4.52)
90 days	4.57 (3.93)	3.61 (3.77)	4.77 (3.85)	5.59 (3.66)	6.30 (3.91)	9.29 (4.12)

*Note*: Other opioids include: Codeine, morphine, buprenorphine, oxycodone, semisynthetic opioid analgesics and synthetic opioid analgesics.

^a^ Age demographics, treatment episode duration and treatment outcomes are *M* (SD). All other values are *n* (%).

### 3.2 Psychological Distress Trajectory Model Fit

Model fit statistics indicated support for all models with five or fewer trajectory classes (see Table 2). All models had more than 1% of the subsample in each class and represented unique trends in the data [38]. Lo–Mendell–Rubin Likelihood Ratio Tests indicated the five‐class model was significantly better than the four‐class model (*p* = 0.023). The six‐class model did not significantly improve fit compared to the five‐class model (*p* = 0.416). Lower Bayesian Information Criterion values supported the five‐class model in comparison to the four‐class model. Correspondingly, the five‐class model was deemed most appropriate.

**TABLE 2:** Goodness of fit indices for the psychological distress trajectory latent growth curve model (*N* = 1492).

Model	Description	BIC	Entropy	BLRT	LMRT	*p*
I	Single trajectory group	33913.161				
II	Two trajectory groups	31534.017	0.864	−16315.222*	1111.50	< 0.001
III	Three trajectory groups	31159.341	0.776	−15734.123*	378.299	0.001
IV	Four trajectory groups	30969.425	0.780	−15535.823*	202.598	< 0.001
**V**	**Five trajectory groups**	**30910.079**	**0.784**	**−15429.904** *	**77.724**	**0.023**
VI	Six trajectory groups	30881.109	0.777	−15389.269*	48.674	0.416

*Note*: The five trajectory group statistics have been bolded to indicate the final selected model.

Abbreviations: BIC, Bayesian Information Criterion; BLRT, Bootstrap Likelihood Ratio; LMRT, Lo–Mendell–Rubin Adjusted Likelihood Ratio Test.

* *p* < 0.001.

### 3.3 Psychological Distress Class Specification

**FIGURE 2:**
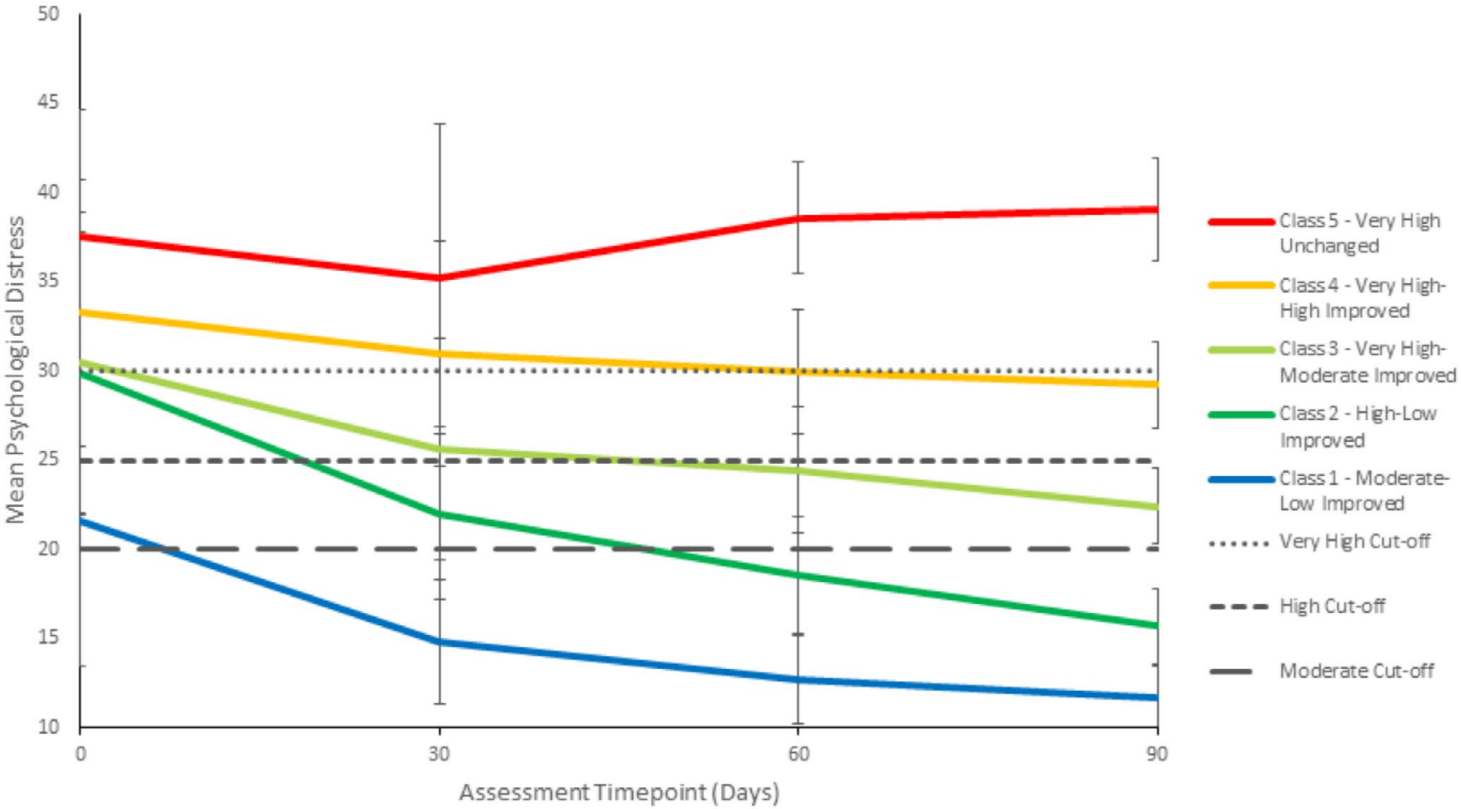
Mean psychological distress (K10 scores) at baseline, 30, 60, 90 days among the five Kessler‐10 Psychological Distress Scale trajectory groups with category bandings (*N* = 1492).

The five classes were labelled based on their patterns of K10 scores over the study period, using the established cut‐offs for clinical categories (see Figure 2) of psychological distress described above [29, 30, 31]. The five groups were Class 1: moderate–low improved, Class 2: high–low improved, Class 3: very high–moderate improved, Class 4: very high–high improved and Class 5: very high unchanged. Classes 1 and 2 demonstrated improvement over time in clinical categories of psychological distress. Class 1 (moderate–low improved) was the largest (*n* = 667; 45.4%), with moderate scores at intake (*M* = 21.58, SD = 8.10) which declined over time to the low clinical category (*M* = 11.72, SD = 1.73) at 90 days. Class 2 (high–low improved; *n* = 515; 34.4%) had high mean scores at intake of 29.88 (SD = 7.88) which declined to the low clinical category (*M* = 15.69, SD = 2.14) at 90 days. Two groups started in the very high psychological distress clinical category but showed improvement over time. Class 3 (very high–moderate improved; *n* = 196; 13.1%) had very high scores at intake (*M* = 30.43, SD = 8.48) which declined to moderate scores at 90 days (*M* = 22.44, SD = 2.12). Class 4 (very high–high improved; *n* = 84; 5.6%) had very high scores at intake (*M* = 33.25, SD = 7.14) that decreased into the high clinical category (*M* = 29.21, SD = 2.43) by 90 days. Class 5 (very high unchanged) was the smallest (*n* = 20; 1.3%), with very high scores at intake of 37.55 (SD = 7.14) that demonstrated no change or improvement from the very high psychological distress over time (All *M* > 30). Both classes 4 and 5 had persistent very high psychological distress at 30 days assessments.

### 3.4 Demographic and Substance Use Correlates of Psychological Distress Classes

The multinomial logistic regression indicated that intake age, described gender, principal drug of concern, reason for treatment cessation, EQoL‐8 and SDS were significant correlates of class membership (see Table 3). For primary substance of concern, heroin and other opioids were reported together as ‘Opioids’ and some categories were omitted for other substance of concern, other gender, or other reason for cessation due to small proportions (*n* = 103). Compared to the high–low improved class (Class 2; *n* = 645, 34.5%; referent group), the moderate–low improved class (1) were more likely to identify as a male (relative risk ratios (RRR) = 1.64 [1.23, 2.19]), have lower SDS (RRR = 0.92 [0.89, 0.96]) and higher EQoL‐8 (RRR = 1.09 [1.07, 1.11]), and were less likely to report cannabis as the primary substance of concern at treatment entry (RRR = 0.54 [0.32, 0.89]). The very high–moderate improved class (3) were more likely to be aged under 25 (RRR = 1.83 [1.11, 3.02]), have lower SDS (RR = 0.93 [0.88, 0.98]) and use opioids (RRR = 2.35 [1.41, 3.94]). The very high–high improved class (4) were less likely to identify as a male (RRR = 0.49 [0.29, 0.82]), and more likely to be aged under 25 (RRR = 2.86 [1.50, 5.47]), have lower EQoL‐8 (RRR = 0.94 [0.90, 0.98]) and lower SDS (RRR = 0.90 [0.84, 0.97]). The very high unchanged class (5) were more likely to have lower EQoL‐8 (RRR = 0.89 [0.82, 0.97]) and less likely to have completed treatment as a reason for cessation (RRR = 0.16 [0.05, 0.49]).

**TABLE 3:** Multinomial regression model examining demographic and clinical correlates associated with trajectory class membership (*N* = 1389, referent category: Class 2 high–low improved; *n* = 475).

	Class 1: Moderate–low improved (*n* = 645)			Class 3: Very high–moderate improved (*n* = 181)			Class 4: Very high–high improved (*n* = 73)			Class 5: Very high unchanged (*n* = 15)		
	RRR	95% CI	*p*	RRR	95% CI	*p*	RRR	95% CI	*p*	RRR	95% CI	*p*
Demographics												
Age, years												
Under 25	0.718	[0.472, 1.093]	0.122	1.826	[1.105, 3.018]	**0.019**	2.858	[1.495, 5.466]	**0.001**	2.499	[0.604, 10.337]	0.206
25 and over	—	—	—	—	—	—	—	—	—	—	—	—
Described gender												
Male	1.641	[1.229, 2.192]	**< 0.001**	0.842	[0.581, 1.222]	0.366	0.488	[0.293, 0.815]	**0.006**	0.779	[0.260, 2.340]	0.657
Female	—	—	—	—	—	—	—	—	—	—	—	—
Substance use												
Principal drug of concern												
Alcohol	—	—	—	—	—	—	—	—	—	—	—	—
Stimulants	1.305	[0.978, 1.741]	0.071	1.341	[0.883, 2.037]	0.169	0.747	[0.407, 1.372]	0.347	0.625	[0.146, 2.670]	0.525
Cannabis	0.536	[0.322, 0.891]	**0.016**	0.852	[0.422, 1.713]	0.652	1.138	[0.492, 1.629]	0.763	1.665	[0.353, 7.846]	0.519
Opioids	1.094	[0.732, 1.636]	0.660	2.352	[1.406, 3.935]	**0.001**	1.242	[0.560, 2.756]	0.594	2.835	[0.642, 12.521]	0.169
Treatment outcomes												
Reason for cessation												
Incomplete	—	—	—	—	—	—	—	—	—	—	—	—
Complete	1.016	[0.688, 1.501]	0.935	0.729	[0.443, 1.200]	0.214	0.681	[0.349, 1.328]	0.259	0.158	[0.051, 0.485]	**0.001**
Quality of Life (EQoL)	1.089	[1.067, 1.112]	**< 0.001**	0.979	[0.952, 1.006]	0.131	0.939	[0.902, 0.977]	**0.002**	0.890	[0.816, 0.971]	**0.009**
Severity of Dependence (SDS)	0.922	[0.888, 0.957]	**< 0.001**	0.931	[0.884, 0.980]	**0.006**	0.903	[0.839, 0.972]	**0.007**	1.055	[0.884, 1.260]	0.551

*Note*: 103 cases were removed due to small proportions for other substances of concern, other described genders and other reasons for cessation. Bolded significance values are significant at *p* < 0.05.

Abbreviations: CI, confidence interval; RRR, relative risk ratios.

### 3.5 Post hoc Treatment Cessation and Duration Analyses

Supporting Information: Appendix B highlights that people who cease treatment for reasons other than completion often leave earlier (median 24 days, range: 9–53) and have higher baseline and 30‐day psychological distress than those who complete treatment. Supporting Information: Appendix B also demonstrates a larger proportion of clients in the COMS sample (10.1%) compared to the participants of the current study (6.9%) would have sustained very high psychological distress at 30 days. This echoes the regression analysis where participants in Class 5 (very high unchanged) were more likely to have earlier treatment exits.

## 4 Discussion

This study used routinely collected data in a large, inclusive sample to identify data‐driven groups based on trajectories of people engaged in residential treatment. Five trajectory groups (Class 1: moderate–low improved; Class 2: high–low improved; Class 3: very high–moderate improved; Class 4: very high–high improved; and Class 5: very high unchanged) were identified based on levels of psychological distress over the first 3 months of residential treatment. Seven percent of the sample (Classes 4 and 5) remained within a high or very high clinical category for psychological distress up to 90 days, despite engaging with treatment. Age, described gender, principal drug of concern, reason for treatment cessation, dependence severity and quality of life at intake were significant correlates of trajectory membership.

The current study extends previous research by identifying distinct groups of individuals based on psychological distress, establishing data‐driven trends and identifying group differences for a range of residential treatment clients. The findings of the current study are consistent with previous studies [17, 19] looking at psychological distress trajectories in treatment, with the majority of the sample (93%; Classes 1–3) demonstrating reductions in psychological distress over the first 3 months of treatment. The trajectories from the current study reflect findings from Chambers et al. [4] where 71% of people had reductions in psychological distress. The current study did not identify the stable low psychological distress class found in Chambers et al. [4]. The two highest classes (Classes 4 and 5; total 7%) in the current study showed limited to no improvement and continued to demonstrate significant psychological distress up to 90 days in treatment. This is a novel finding compared to previous studies demonstrating varying improvement for all groups in the first 3 months after treatment entry [4, 17, 19]. The unique identification of non‐improving groups in the current study may be due to the larger sample, naturalistic treatment data, or broader inclusion criteria in this study. This finding has enabled further exploration of a group that did not improve and is likely at greater risk of treatment cessation for reasons other than completion [7, 8].

Participants from the highest trajectory classes (4 and 5) were more likely to be under 25 years old, identify as female, have lower quality of life and dependence severity at baseline, and for the highest class were more likely to leave treatment for other reasons. Moreover, men were significantly more likely to be in the lower trajectories with greater psychological distress decreases. Consistent with existing literature [40, 41] and literature highlighting poorer outcomes for women [42] and young people [43], these findings indicate that more research evaluating outcomes for women and young people in substance use treatment is needed to further improve their treatment outcomes. Psychological distress may be higher for these groups both at treatment entry and throughout a treatment episode due to age‐ and gender‐related differences in psychological distress, treatment needs of clients and barriers to treatment access [44, 45, 46, 47, 48, 49, 50].

Quality of life and psychological distress are negatively correlated treatment outcomes [5], and it is unsurprising that with higher psychological distress, trajectories were associated with lower baseline quality of life scores. Baseline dependence severity was highest in the referent category (high–low improved). The very high unchanged class showed little decrease in dependence severity across all four assessment timepoints, and by 90 days, all other classes showed similar decreasing trends between dependence and psychological distress severity at the same point. Future research exploring the relationship between dependence severity and psychological distress over time in treatment, potentially using linear mixed models, would be beneficial here.

Regarding primary substance of concern, the moderate–low improved class were less likely to use cannabis compared to the high–low improved class. By contrast, reporting opioids as the primary substance of concern was a significant predictor of the very high–moderate improving class. The current study did not control for additional pharmacological interventions delivered during the residential treatment episodes; however, the use of pharmacological interventions in conjunction with residential treatment may have contributed to this improvement. Integrated and concurrent treatment types designed to meet the needs of the individual are widely recommended [14]. Further studies examining the combination of opioid agonist treatment and residential treatment episodes on treatment outcomes for people reporting opioids as the primary substance of concern are needed.

This study provides beneficial insights into the trajectories of psychological distress during a residential treatment episode. The large sample, naturalistic client data and use of three timepoints for the growth analyses ensured statistical rigour in examining trajectories and enabled the identification of a novel subgroup who were not improving over the first 90 days of treatment. The samples used for both analyses are more inclusive than previous studies, including both men and women, from more varied service types and providers. However, there are limitations with the study. This data was grouped into timepoints based on days from admission to form intake, 30, 60 and 90 days assessments. The 90‐day assessment was selected as the final timepoint for statistical reasons due to missingness, sample size and statistical power. A maximum missingness of 90% between two timepoints was set as the threshold to ensure model convergence, and a minimum of three timepoints are needed to evaluate curvature of the trends [37, 51]. Assessment completion at later timepoints greatly decreased, likely due to treatment exit or service characteristics around when assessments are completed, prompting the exclusion of many participants. This decrease also had consequent effects on the statistical power in identifying classes. Typically, more participants are required to correctly identify models with greater complexity or fewer observations [52] to avoid overspecification of the model. As such, the 90‐day assessment was selected as the most inclusive timepoint for the study. Moreover, these arbitrary timepoints reflect a period +/−15 days from the stated assessment day. This should be taken into consideration when generalising intake characteristics as they may not be reflective of true baseline values but rather values after a maximum of 14 days in treatment. It is important to recognise that the subsample was restricted and reflects an adult population who engaged in residential AOD treatment for at least 45–74 days and attended services that routinely collect outcome measures in the first 3 months of treatment. However, sensitivity analyses indicate this sample was largely comparable to the broader population of clients attending residential substance use disorder treatment in NSW.

It is likely that the number of participants experiencing high psychological distress over time in treatment and post‐discharge is higher than demonstrated here. This is supported by prior literature in which high psychological distress is related to an increased likelihood of leaving treatment before completion [7, 8]. Considering Supporting Information: Appendix B, it is likely people attending treatment may have discharged prior to completing three K10 timepoints and would not have been included in the current study. As there was inadequate post‐treatment follow‐up data, these results need to be considered as reflecting within‐treatment episode outcomes.

The data used in this study was from a broader routinely collected database from clients in treatment. It is unclear the extent to which individual clinicians use the results of these assessment measures to inform care. Evidence suggests that using routinely collected data across a treatment episode in reviewing treatment planning improves outcomes for clients, including increased treatment retention, global functioning and improvements for mood symptoms [53, 54, 55]. Routine collection of outcome measures is beneficial for identifying clients who may not be responding to treatment [56] and enables more targeted or focused individualised care to be implemented, in line with measurement‐based care frameworks [57]. Implementing the types of trajectories shown here into client management systems prompts clinicians to engage further with outcome measurement. For example, if clinicians are informed that their clients have poorer trajectories, they can use this information to alter or adjust treatment earlier, in consultation with clients to capture their needs and improve desired treatment outcomes. This has been implemented in other substance use and psychotherapy outcome management systems and has substantially improved treatment effectiveness for clients [58, 59].

Generally, measurement‐based care has been widely explored in the mental health literature [57] but requires a richer evaluation and implementation for substance use disorders. Future research identifying optimal cut‐points on measures, exploring the optimal frequency of administration for routine outcome measures for early intervention, and evaluation of group trajectories for other measures and treatment types is needed across the AOD sector. In addition, future studies examining the effectiveness of additional interventions for clients identified as having high psychological distress are needed.

## 5 Conclusions

Among this cohort with strong treatment engagement, there are distinct psychological distress trajectories during residential treatment. Although psychological distress improved across the first 3 months of residential treatment for the majority, around 7% reported sustained high levels of psychological distress. This group is at greater risk of poorer treatment outcomes. Higher levels of psychological distress were associated with being younger, identifying as female, having lower quality of life and reporting higher levels of dependence severity. At an individual client level, routine outcome monitoring represents an important opportunity to identify non‐improving clients, review the treatment plan and provide shared treatment options for additional interventions and/or supports. At a service level, these findings highlight the need for integrated, shared or specialised care for younger people and women to provide more intensive support.

## Author Contributions

Each author certifies that their contribution to this work meets the standards of the International Committee of Medical Journal Editors.

## Conflicts of Interest

This research is partially supported by an Australian Research Training Program Scholarship for E.L.H. This work was supported by the National Health and Medical Research Council Meaningful Outcomes in Substance Use Treatment Centre of Research Excellence. Authors M.L.L., R.S. and M.C. are employed by the Network of Alcohol and Other Drugs Agencies (NADA). B.L., L.R. and P.J.K. have previously received research consultancies from NADA.

## Supporting information


Data S1.

## Data Availability

The data that support the findings of this study are available from NADA. Restrictions apply to the availability of these data, which were used under license for this study. Data are available from the author(s) with the permission of NADA.
