# Supplementary material for: Psychological Distress Trajectories in Residential Alcohol and Other Drug Treatment
Source: Drug Alcohol Rev. 2025 Jun 17;44(5):1308–20. doi: 10.1111/dar.14099 (PMC12228035; doi:10.1111/dar.14099)
Supplement: Supplementary file 1 — Data S1. [file DAR-44-1308-s001.docx]

**Supplementary Material**

**Psychological Distress Trajectories in Residential Alcohol and Other Drug Treatment**

Emma L. Hatton^1^, Peter J. Kelly^1^, Laura Robinson^1^, Alison Beck^1^, Mei L. Lee^2^, Robert Stirling^2,3^, Lauren Mullaney^4^, Michele Campbell^2^, & Briony Larance^1^

**Affiliations:**

1. *School of Psychology, University of Wollongong, Australia*
2. *Network of Alcohol and Other Drug Agencies, Sydney, Australia*
3. *Drug Policy Modelling Program, Social Policy Research Centre, UNSW Sydney, Sydney, Australia*
4. *Triple Care Farm, Mission Australia, Knights Hill, Australia*

**Appendix A**

*Table A1. Comparison of characteristics of trajectory sample participants with all other residential treatment clients in NADABase*

|  | | Final trajectory sample (three or more COMS assessments)  (*N* = 1492) | | | All other residential treatment clients  (*N* = 25,476) | | |
| --- | --- | --- | --- | --- | --- | --- | --- |
|  | | *n* | % | 95% CI | *n* | % | 95% CI |
| **Demographic characteristics** | |  |  |  |  |  |  |
| Described gender | |  |  |  |  |  |  |
|  | Men | 1068 | 71.6 | [69.3, 73.9] | 17080 | 67.0 | [66.4, 67.6] |
|  | Women | 409 | 27.4 | [25.1, 29.7] | 7978 | 31.3 | [30.7, 31.9] |
|  | Other | 15 | 1.0 | [0.5, 1.5] | 418 | 1.6 | [1.4, 1.8] |
| Age, years^ | | 36.72 | 9.60 | [36.23, 37.21] | 36.54 | 11.17 | [36.40, 36.68] |
| Country of birth | |  |  |  |  |  |  |
|  | Australia | 1346 | 90.2 | [88.7, 91.7] | 23124 | 90.8 | [90.4, 91.2] |
|  | Other | 146 | 9.8 | [8.3, 11.3] | 2352 | 9.2 | [8.8, 9.6] |
| Usual Accommodation | |  |  |  |  |  |  |
|  | Stable housing^b^ | 1163 | 77.9 | [75.8, 80] | 17364 | 68.2 | [67.6, 68.8] |
|  | Unstable housing^c^ | 217 | 14.5 | [12.7, 16.3] | 12.7 | 12.7 | [12.3, 13.1] |
|  | Prison or detention centre | 28 | 1.9 | [1.2, 2.6] | 1103 | 4.3 | [4.1, 4.5] |
|  | Treatment facility/hospital | 44 | 2.9 | [2.0, 3.8] | 750 | 2.9 | [2.7, 3.1] |
|  | Other/not stated | 40 | 2.7 | [1.9, 3.5] | 3019 | 11.9 | [11.5, 12.3] |
| **Substance use** | |  |  |  |  |  |  |
| Principal drug of concern | |  |  |  |  |  |  |
|  | Alcohol | 516 | 34.6 | [32.2, 37.0] | 9073 | 35.6 | [35.0, 36.2] |
|  | Stimulants | 621 | 41.6 | [39.1, 44.1] | 9637 | 37.8 | [37.2, 38.4] |
|  | Cannabis | 118 | 7.9 | [6.5, 9.3] | 3261 | 12.8 | [12.4, 13.2] |
|  | Heroin | 171 | 11.5 | [9.9, 13.1] | 2181 | 8.6 | [8.3, 8.9] |
|  | Other opioids | 33 | 2.2 | [1.5, 2.9] | 743 | 2.9 | [2.7, 3.1] |
|  | Other/unknown | 33 | 2.2 | [1.5, 2.9] | 581 | 2.3 | [2.1, 2.5] |
| **Clinical profile at treatment entry** | |  |  |  |  |  |  |
| Psychological Distress (K10)^ | | 26.47 | 9.24 | [26.00, 26.94] | 26.52 | 9.42 | [26.40, 26.64] |
| Quality of Life (EQoL-8)^ | | 25.17 | 6.72 | [24.83, 25.51] | 24.81 | 6.57 | [24.73, 24.89] |
| Severity of Dependence (SDS)^ | | 9.49 | 3.68 | [9.30, 9.68] | 9.09 | 3.67 | [9.05, 9.14] |

*Note.*  ^a^Percentage of the total. ^b^Stable housing included rented house or flat, privately owned houses or flats. ^c^Unstable housing included boarding houses, hostels or supported accommodation services, shelters, refuges, and caravans. ^Age demographics are M(SD). Other opioids includes codeine, morphine, buprenorphine, oxycodone, semisynthetic opioid analgesics and synthetic opioid analgesics.

CI, confidence interval; COMS, Client Outcomes Management System; K10, EQoL-8, EUROHIS Quality of Life scale; Kessler-10 Psychological Distress Scale; SDS, Severity of Dependence Scale.

**Appendix B**

Descriptive statistics, proportions, t-tests and chi-squared tests were conducted in SPSS Version 25 using the total Client Outcomes Management System (COMS) sample (*n* = 10,021) to explore differences in psychological distress and treatment duration for clients who completed treatment compared (*n =* 4.542) to clients who left treatment for other reasons (*n =* 4258). Clients whose treatment episodes ended due to death, legal intervention, moving out of area, or other unknown reasons were excluded from the analysis (*N* = 1221). Participants who were identified as having completed their treatment episode (complete or transferred to another service) were compared to participants who were identified as having left treatment for other reasons (left without notice, left against advice, or left involuntarily). In the COMS sample, participants who completed treatment, the total median time in treatment was 54 days longer than those who had left for other reasons (Median = 76, IQR = 28-140; versus Median = 24, IQR 9-53). Moreover, baseline psychological distress was significantly lower (t = -7.243, p <0.001) for COMS clients who had completed treatment (M = 25.81, SD = 9.21) compared to COMS clients who left treatment for other reasons (M = 27.36, SD = 9.46). A crosstabulation using chi-square statistics also supported that clients who had very high psychological distress scores (>30) at both baseline and 30 day assessments compared to clients who did not have very high psychological distress scores at either or both of the baseline and 30 day assessments but who completed both assessments, were more likely to have left for other reasons$\boldsymbol{\chi}^{\boldsymbol{2}}$ treatment episode ($\boldsymbol{\chi}^{\boldsymbol{2}}$= 4.50, df = 1, *p* = 0.036).

When comparing between the total COMS sample and the final trajectory sample included in the study (*n* = 1492), there were also a larger proportion of these clients who left for other reasons before completing treatment in the COMS sample (10.1%) than in the participants of the current study who comprised the Very High Unchanged and Very High-Improved Classes (6.9%).

Considering these results, there is support for ~24 days (range 9-53) as a key risk point for early, unplanned treatment exits from residential treatment, and prolonged very high psychological distress (K10 score > 30) as a risk-factor drop out.

*Table B1. Comparison of median time in treatment and psychological distress for Client Outcomes Management System clients who were identified as having completed treatment and those who left treatment for other reasons to identify key risk points for exiting treatment.*

|  |  | Completed treatment  (n = 4542) | | Left treatment for other reasons (n = 4258) | | Comparison | |
| --- | --- | --- | --- | --- | --- | --- | --- |
|  |  | *Median* | *IQR* | *Median* | *IQR* | *U* | *p* |
| Time in treatment | | 76 | 28-140 | 24 | 9-53 | 6184158.00 | <.001 |
|  |  |  | *SD* | *M* | *SD* | *t* | *p* |
| Psychological distress | |  |  |  |  |  |  |
|  | Baseline | 25.81 | 9.21 | 27.36 | 9.46 | -7.243 | < 0.001 |
|  | 30 days | 19.83 | 6.75 | 21.34 | 8.09 | -6.009 | < 0.001 |
|  | 60 days | 17.34 | 6.44 | 20.40 | 7.91 | -6.864 | < 0.001 |
|  | 90 days | 16.10 | 5.86 | 20.24 | 7.50 | -7.344 | < 0.001 |
|  |  | *n* | *%* | *n* | *%* | $\boldsymbol{\chi}^{\boldsymbol{2}}$ | *p* |
| Sustained psychological distress classification between baseline and 30 days* | |  |  |  |  | 4.50 | 0.036 |
|  | Very high | 161 | 3.5 | 101 | 2.4 |  |  |
|  | High, moderate or low | 2081 | 45.8 | 913 | 21.4 |  |  |
|  | Missing/unknown | 2300 | 50.6 | 3244 | 76.2 |  |  |

*Note.* * Sustained psychological distress classification between baseline and 30 days was defined as treated as a binary variable for clients who had total psychological distress scores greater than 30 (very high) at both baseline and 30 days compared to all other participants.

IQR, interquartile range.
